# Interactions and Curing Dynamics Between UV-Triggered Epoxy Acrylate Binder, Curing Agents and Photoinitiators

**DOI:** 10.3390/polym17091252

**Published:** 2025-05-04

**Authors:** Ji-min Choi, Sang Jang, Keon-Soo Jang

**Affiliations:** Department of Polymer Engineering, School of Chemical and Materials Engineering, The University of Suwon, Hwaseong 18323, Gyeonggi-do, Republic of Korea

**Keywords:** epoxy acrylate, photoinitiator, UV curing, curing agents

## Abstract

This study investigated the interaction between UV-triggered curing binders and photoinitiators, focusing on their thermal, mechanical, and morphological properties. Using epoxy acrylate as the matrix and three potential photoinitiators with varying phosphorus contents, UV curing systems were fabricated and analyzed. 2-hydroxy-2-methyl-1-phenyl-1-propanone (HMPP), 2,4,6-trimethyl benzoyl diphenyl phosphine oxide (TPO), and their mixture were utilized as photoinitiators. We observed that the curing process significantly reduced residual double bonds within the first 5 s of UV irradiation time. The glass transition temperature (T_g_) increased with curing time due to enhanced network density. For instance, in the MyA–TPO formulation, T_g_ of the cured sample tended to increase to 67.3 °C for 3 s to 79.8 °C for 15 s. Mechanical analysis revealed that HMPP facilitated the formation of robust network structures. Notably, the MyA–HMPP formulation exhibited a tensile strength of 63 MPa and a Young’s modulus of 21 MPa, indicating excellent mechanical strength. SEM imaging confirmed these findings, illustrating distinct fracture morphologies that correlated with mechanical performance. These results provide insights into optimizing UV-curable materials for applications requiring high precision and durability. In particular, the combination of high T_g_, superior tensile strength, and uniform fracture morphology indicates excellent thermal stability, mechanical integrity, and crack resistance—critical requirements in semiconductor packaging. These properties, along with rapid UV curing, support the suitability of the proposed systems for advanced applications such as system-in-package (SiP) and 3D integration.

## 1. Introduction

Photocuring technology, which involves the use of light to initiate chemical reactions that lead to the solidification of materials, has undergone remarkable advancements in recent decades. The technology has been extensively utilized in various industries, including electronics, coatings, adhesives, and even biomedicine [1,2]. By replacing traditional thermal curing methods, photocuring has provided rapid, efficient, and environmentally friendly manufacturing processes [3,4,5,6]. Compared to thermal curing systems, photocuring achieves solidification in significantly shorter timescales, leading to enhanced productivity and energy efficiency [7,8]. Such benefits have been made possible by using suitable photoinitiators, resin formulations, and light sources, making photocuring effective [9,10].

The origins of photocuring can be traced back to the 1960s when ultraviolet (UV) curing systems were first introduced for industrial applications, primarily in coatings and printing. Over the decades, the development of photoinitiators, such as free-radical and cationic systems, allowed the technology to extend its reach to other industries. Photocuring involves the use of photoinitiators that absorb light, typically in the UV or visible spectrum, and generate reactive species, such as free radicals or cations [11]. These reactive species initiate polymerization reactions, converting liquid resins into hard polymers. The rapid reaction kinetics and spatial control offered by photocuring make it highly desirable for precision applications.

Among the many applications of photocuring, its role in the semiconductor industry is particularly noteworthy [12,13]. In semiconductor manufacturing, photocuring enables the formation of high-resolution, precise patterns essential for microelectronics [14,15,16]. This capability is critical in processes such as photolithography, where UV light is used to define circuit patterns on silicon wafers [17]. Beyond lithography, photocurable adhesives play a vital role in chip packaging, providing robust bonding and insulation in multilayer architectures [18,19,20,21,22]. For instance, UV-curable adhesives ensure stable attachment between semiconductor chips and substrates while offering excellent thermal and electrical insulation properties [23,24]. The ability to achieve rapid curing under controlled conditions has also made photocuring indispensable for advanced packaging techniques, such as system-in-package (SiP) and 3D integration [25,26,27].

Despite its advantages, photocuring technology features some drawbacks. A key limitation is its dependence on UV light, which struggles to penetrate thick or opaque materials, leading to incomplete or uneven curing [28]. This is particularly problematic in applications requiring high mechanical strength and durability [29]. Moreover, cured materials may exhibit suboptimal impact resistance or crack propagation resistance, requiring additional enhancements [30]. Environmental factors, such as temperature and humidity, also significantly influence the curing process and final properties of photocured materials [31,32]. These challenges require careful formulation and system design to optimize performance under various conditions.

Recent progress in photoinitiator chemistry has been instrumental in addressing these challenges. For example, free-radical photoinitiators, which generate radicals upon UV exposure, have been optimized for high reactivity and efficiency. Similarly, cationic photoinitiators, which enable the polymerization of epoxies and vinyl ethers, offer advantages, such as reduced oxygen inhibition and greater thermal stability. In resin formulations, the introduction of multifunctional acrylates and hybrid systems combining epoxy and acrylate chemistries has enabled the development of materials with improved mechanical properties, chemical resistance, and crosslink density.

Previous studies on UV-curable systems using conventional free-radical and cationic photoinitiators have reported moderate thermal and curing performances. T_g_ values of approximately 60 °C have been reported in epoxy acrylate/methacrylate systems cured with typical free-radical initiators [33]. Similarly, cationic initiators have shown advantages in oxygen tolerance and depth of cure but typically require longer curing times—ranging from 15 to 30 s under standard UV conditions [34].

In contrast, the systems investigated in this study, particularly those utilizing phosphorus-containing photoinitiators such as TPO, exhibited T_g_ values exceeding 70 °C and achieved effective curing within 5 s. These enhancements are attributed to the high radical generation efficiency and rapid network formation enabled by phosphorus functionality.

Such comparisons highlight the need for a more comprehensive understanding of how phosphorus-containing photoinitiators influence not only photoreactivity but also thermal and mechanical performance. This study addresses that gap through a systematic evaluation of curing behavior and structure–property relationships in UV-curable epoxy acrylate systems with varying phosphorus content.

This study builds upon these advancements by investigating the interaction between UV-curable binders and photoinitiators, focusing on the role of phosphorus-containing photoinitiators [35]. Phosphorus is known to impart unique properties to photoinitiators, such as flame retardancy and enhanced reactivity. However, curing dynamics and their impact on UV-cured bulk systems are still unknown. Previous studies have mainly focused on material design or surface-level curing, but insight into the impact of phosphorus content on network formation, thermal stability, and mechanical strength of bulk systems is lacking [36,37]. To explore these effects, three photoinitiators with varying phosphorus content (100%, 50%, and 0%) were selected, and their impact on the physical and chemical properties of epoxy-acrylate systems was analyzed.

The findings of this study are expected to provide valuable insights into the design and optimization of photocurable materials for demanding applications. While the current investigation focuses on specific formulations and UV curing conditions, future research should explore the broader implications of varying light sources, such as LED and laser systems, and the integration of novel additives to enhance long-term durability and environmental stability. By addressing these challenges, photocuring technology can continue to expand its applications and meet the evolving demands of modern industries.

## 2. Experimental Section

### 2.1. Materials

**Figure 1:**
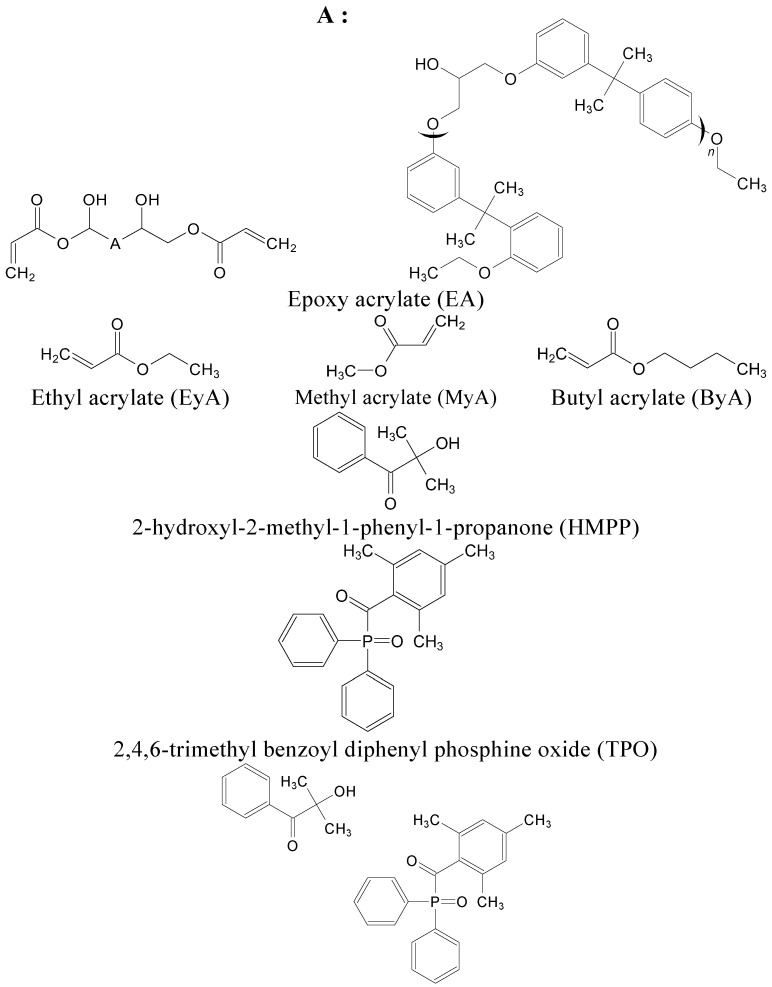
Materials used in this study.

To fabricate the UV curing system, epoxy acrylate (EA, Profinechem Co., Republic of Korea) with a molecular weight of 484.5 g/mol was used as a matrix. Ethyl acrylate (EyA, 99.5%), methyl acrylate (MyA, 99.8%), and butyl acrylate (ByA, 99.0%) as acylate-based curing agents were purchased from Samchun Pure Chemicals Co., Ltd., (Republic of Korea). Potential photoinitiators such as 2-hydroxy-2-methyl-1-phenyl-1-propanone (HMPP, Profinechem Co., Republic of Korea) and 2,4,6-trimethyl benzoyl diphenyl phosphine oxide (TPO, Profinechem Co., Republic of Korea) were used. In addition, a mixture of the two photoinitiators, HMPP and TPO, is denoted by MIX in this study. Figure 1 presents the materials used in this study.

### 2.2. Fabrication of UV Curing Pastes and UV-Cured Sample

**Figure 2:**
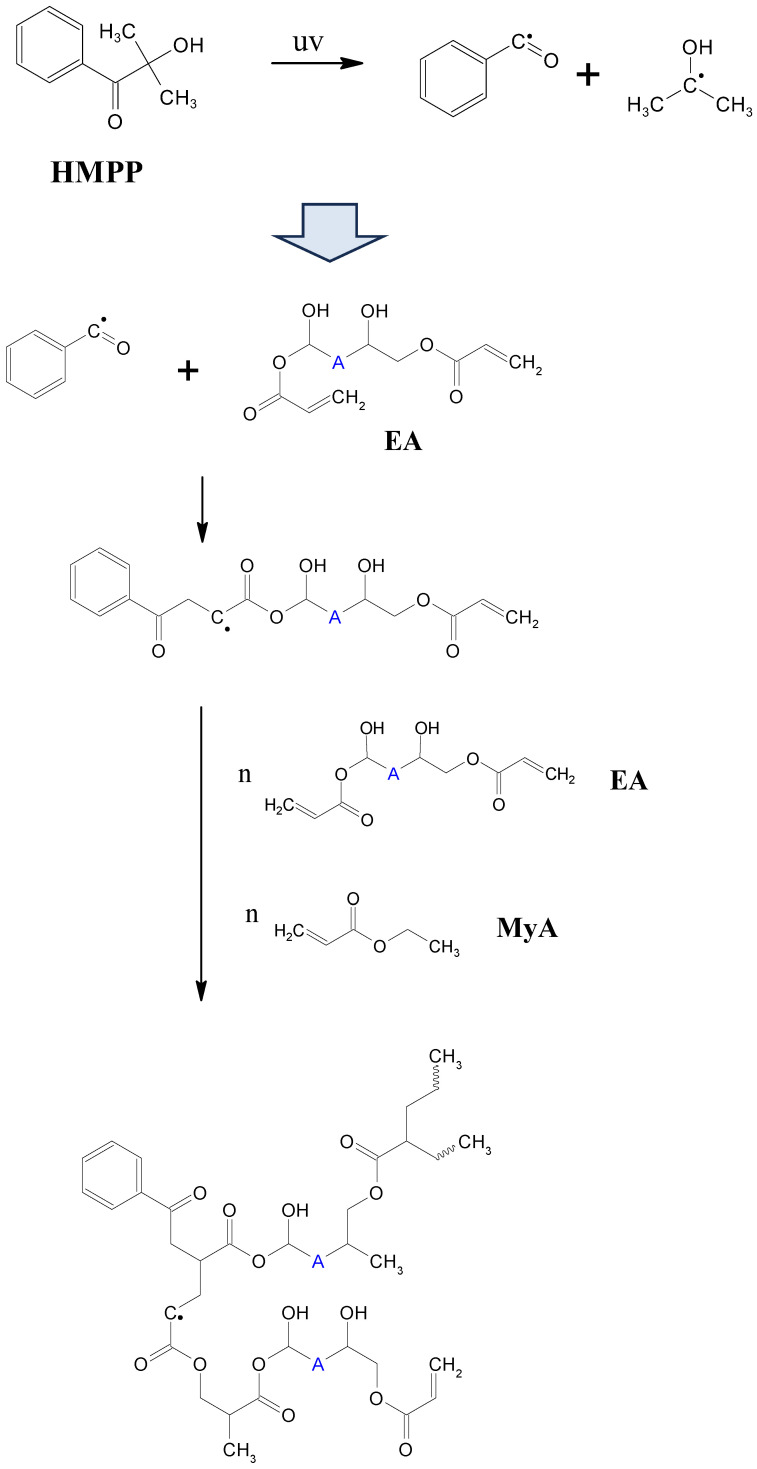
Reaction mechanism of UV curing.

The epoxy acrylate and curing agent were mixed in an equivalent ratio of 1:1 to ensure optimal curing. A photoinitiator was added at a concentration of 1 phr relative to the total amount of epoxy resin, as shown in Table 1. Due to the liquid nature of the curing agent, no additional solvents were needed to achieve effective dispersion. The components were thoroughly mixed in a 15 mL vial using a vortex mixer for approximately 10 min to ensure uniform distribution. Following mixing, the homogeneous solution was immediately poured onto a silicone plate, and the UV curing process was promptly initiated as shown in Figure 2.

### 2.3. Curing of Epoxy Acrylate-Based Binder

**Figure 3:**
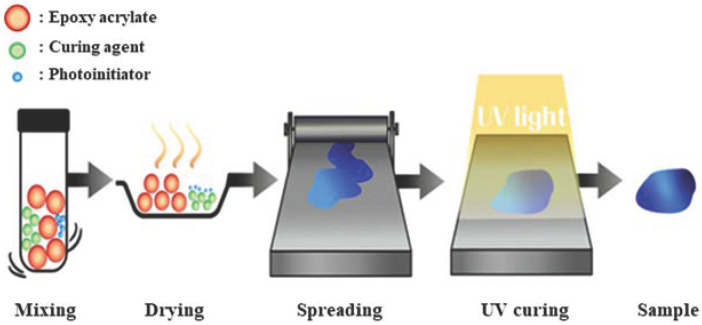
Fabrication of UV curing pastes and cured sample.

The epoxy acrylate and curing agent were mixed at an equivalent ratio of 1:1. A photoinitiator was incorporated at a concentration of 1 wt% relative to the epoxy acrylate matrix. To achieve uniform mixing of the UV paste, a vortex mixer was utilized for approximately 30 min. Following this, as illustrated in Figure 3, a doctor blade was used to spread the paste into a film with a uniform thickness of 3 mm on a silicone plate, ensuring consistent light exposure. The UV curing process was conducted by irradiating the samples with 100% UV intensity for durations ranging from 0 to 10 s. The physical properties of the samples were evaluated in their pre-cured, fully cured, and partially cured states. After curing, the samples were cooled to room temperature (22–24 °C) prior to being carefully removed from the silicone plate.

### 2.4. Characterization

#### 2.4.1. UV Curing

The UV curing of samples was carried out using an INTELLI-RAY 600 system (Uvitron International Inc., West Springfield, MA, USA). Samples were prepared by spreading the UV paste into a film on a silicone rubber plate (30 cm × 30 cm × 5 mm) using a doctor blade. The curing experiments were conducted at a distance of 177.8 mm from the light source. Preliminary tests indicated that complete curing was achieved within 5 s, so curing times were fixed at 0, 3, 5, and 10 s for further analysis.

#### 2.4.2. Thermal Properties

Modulated differential scanning calorimetry (mDSC) was utilized to evaluate the thermal properties of the samples. In contrast to conventional DSC, mDSC introduces a modulation signal to the linear temperature changes, enabling a more precise determination of the glass transition temperature (T_g_). The experiments were conducted under a nitrogen purge at a flow rate of 50 mL/min. The temperature was increased from 0 °C to 200 °C at a scanning rate of 2 °C /min, with a modulation of 1 °C applied every 120 s. Approximately 2 mg of UV paste, prepared with varying photoinitiator compositions, was sampled and placed in aluminum hermetic pans for thermal analysis. The T_g_ of the cured samples was measured as a function of curing time from 0 s to 30 s.

#### 2.4.3. Curing Properties

Fourier transform infrared (FTIR) spectroscopy (Spectrum Two, PerkinElmer Inc., Waltham, MA, USA) was employed to evaluate the changes in bonding peaks during and after the curing process of the epoxy acrylate-based UV curing system. Infrared spectra were recorded across the range of 4000 cm^−1^ to 650 cm^−1^. Samples were subjected to curing times of 0, 3, 5, and 10 s to analyze the progression of the curing process and the bonding energy after completion. This method facilitated detailed peak analysis and the monitoring of curing completion.

#### 2.4.4. Mechanical Properties

Dynamic mechanical analysis (DMA; Model DMA850, TA Instruments Inc., New Castle, DE, USA) was performed to evaluate the mechanical properties of the samples in tensile mode. Rectangular specimens with dimensions of 10 mm in width and 4 mm in thickness were prepared. The storage modulus, loss modulus, and tan δ were measured. The glass transition temperature (T_g_) was determined based on the peak of tan δ. Measurements were conducted at a single frequency of 1 Hz with a constant displacement of 30 μm, using a heating rate of 3 °C/min across a temperature range of 0 °C to 100 °C. In addition, tensile strength tests were carried out on fully cured samples using a universal testing machine (UTM; LR10K Plus, Lloyd Instruments, AMETEK Inc., Berwyn, PA, USA). The tests were performed on specimens with a length of 35 mm, thickness of 2 mm, and width of 10 mm. The gauge length of the samples was maintained at 15 mm, and tests were conducted at room temperature (22–24 °C) with a crosshead speed of 10 mm/min.

#### 2.4.5. Morphology

The fracture surfaces of the UV-cured samples were examined using scanning electron microscopy (SEM; Apreo, FEI Co., Hillsboro, OR, USA) to assess the curing morphology and uniformity of the fractured surfaces, which are indicative of mechanical properties. Samples from tensile tests were prepared by cutting them into dimensions of 2 cm × 0.7 cm × 0.5 cm and mounting them on carbon tape. The surfaces were sputter-coated with gold for 60 s prior to imaging. SEM measurements were conducted in Everhart–Thornley Detector (ETD) mode under an operating voltage of 10 kV, a current of 0.8 nA, and a spot size of 11.0, with images captured at a magnification of 1000×.

## 3. Results and Discussion

### 3.1. Fabrication of UV Curing Pastes and Cured UV Sample

**Figure 4:**
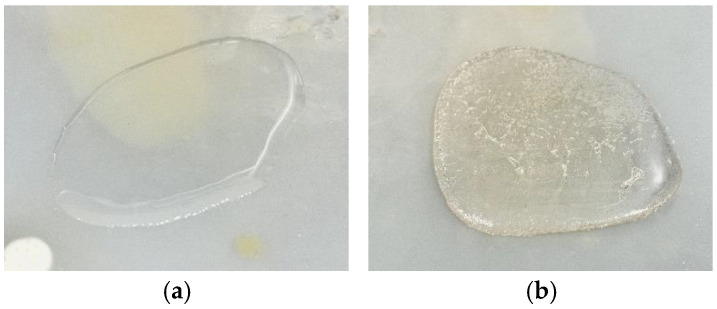
Samples after 10 s of UV irradiation: (**a**) epoxy acrylate; (**b**) epoxy acrylate with curing agent (EyA) and photoinitiator (HMPP).

The photoinitiator concentration was maintained at 1 phr relative to the epoxy acrylate matrix, with the UV lamp intensity set to 100%. Figure 4a illustrates a sample of epoxy acrylate binder without any curing agent subjected to 10 s of UV curing. As shown, in the absence of curing agents, the epoxy acrylate remained in a liquid state with low viscosity, indicating that it lacks sufficient reactive functionality to form a solid network on its own. In contrast, Figure 4b shows the result of curing epoxy acrylate with EyA as a curing agent and HMPP as a photoinitiator under identical conditions. This sample became a fully cured, rigid material, demonstrating that the presence of both a multifunctional acrylate curing agent and a suitable photoinitiator is essential for initiating and completing the crosslinking reaction via free-radical polymerization.

**Figure 5:**
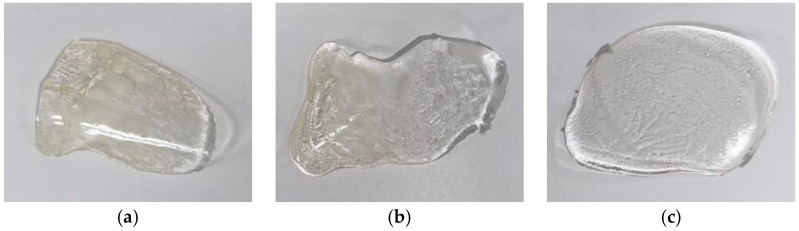
Samples after 10 s of UV irradiation with photoinitiator (TPO): (**a**) MyA, (**b**) EyA, and (**c**) ByA.

Figure 5 further compares the curing behavior using TPO as the photoinitiator and three different acrylate-based curing agents: MyA, EyA, and ByA. While all samples generally hardened after 10 s of UV exposure, the sample containing ByA displayed visibly lower hardness and exhibited residual uncured material on the surface. This incomplete curing behavior is attributed to the structural characteristics of ByA. Its long butyl side chain imparts greater molecular flexibility, which not only reduces the overall reactivity and mobility of radicals during polymerization but also lowers the crosslinking density of the cured network. The inherent flexibility of ByA-based systems limits their suitability for applications requiring long-term durability and high stress resistance; however, it makes them well-suited for applications that demand flexibility, stretchability, impact absorption, and foldability, such as deformable coatings, soft adhesives, and protective layers subjected to dynamic mechanical loads. In contrast, MyA and EyA have shorter alkyl chains, which enhance their reactivity and compatibility with the epoxy acrylate matrix, resulting in more efficient and uniform curing. In addition, the fractured surfaces of the cured samples were examined, as presented in Figure S1 (Supplementary Information), and exhibited minimal differences across all samples.

### 3.2. Thermal Properties of UV Paste

**Figure 6:**
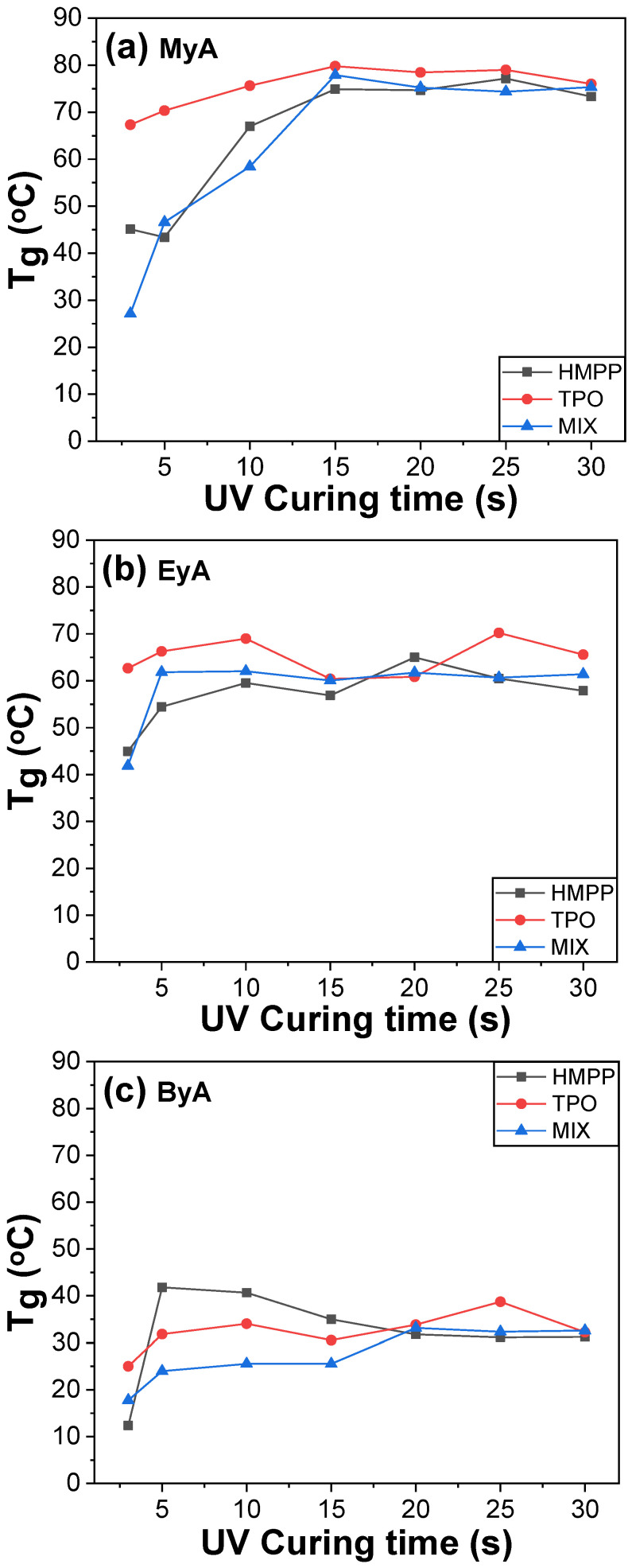
DSC scans of UV-cured samples produced with different curing agents and photoinitiators as a function of UV curing time.

The thermal properties of photocured materials are important. The T_g_ values of the sample containing ByA were consistently lower than those observed with the combination of EyA and MyA, as shown in Figure 6. This behavior is attributed to the long alkyl chain structure of ByA, which imparts high chain flexibility and acts as an internal plasticizer. The increased segmental mobility leads to reduced network density and intermolecular interactions, thereby reducing the T_g_. As curing time increased from 3 to 10 s, a consistent rise in T_g_ was observed for all formulations and reached the saturation point for most samples. This behavior can be explained by the progressive consumption of unreacted C=C double bonds, resulting in increased crosslink density. Higher crosslink density limits molecular motion, thereby enhancing the thermal and mechanical properties of the cured material. However, the T_g_ of the MyA-based samples slightly increased up to 15 s of the UV irradiation, likely because of reduced steric hindrance associated with the short chain of MyA during curing. The T_g_ of all cured systems exhibited minimal change, suggesting a competitive interplay between UV-induced curing and thermal or photodegradation beyond 10 s of UV irradiation.

The DSC results represent the thermal properties of the samples. As shown in Figure 6c, ByA, which possesses a long alkyl chain, exhibits higher flexibility compared to other curing agents, potentially leading to a reduction in network density. This flexibility is also related to the mechanical properties evaluated by UTM. As will be observed in mechanical properties, the high flexibility of ByA resulted in significantly lower mechanical performance, with the tensile strength being particularly low. Furthermore, the DMA analysis, which examined both thermal and mechanical properties, revealed T_g_ values similar to those from DSC. However, the rapid reactivity of the TPO photoinitiator and excessive crosslinking observed in the UTM experiments negatively affected the mechanical strength, which was also reflected in the DMA results. Specifically, the excessive reactivity of TPO led to a heterogeneous network structure, resulting in a relatively lower T_g_ value, as will be seen in the DMA results.

Photocuring systems incorporating TPO as a photoinitiator generally demonstrated superior thermal performance, showing higher T_g_ values across different curing agent systems. This is likely due to TPO’s high radical generation efficiency, which promotes deeper and more uniform curing through rapid initiation. However, the rapid and aggressive curing behavior of TPO may also lead to excessive or uneven crosslinking, particularly near the surface, which can cause local inhomogeneities in the network. This effect is reflected in the DMA results, where samples cured with TPO exhibited lower T_g_ values compared to those cured with other photoinitiators. The inconsistency between DSC and DMA results suggests that the rapid reaction kinetics of TPO may create a heterogeneous network structure, limiting overall mechanical strength and reducing the effective T_g_ under dynamic mechanical loading.

### 3.3. FT-IR Analysis of UV Paste as a Function of Curing Time

**Figure 7:**
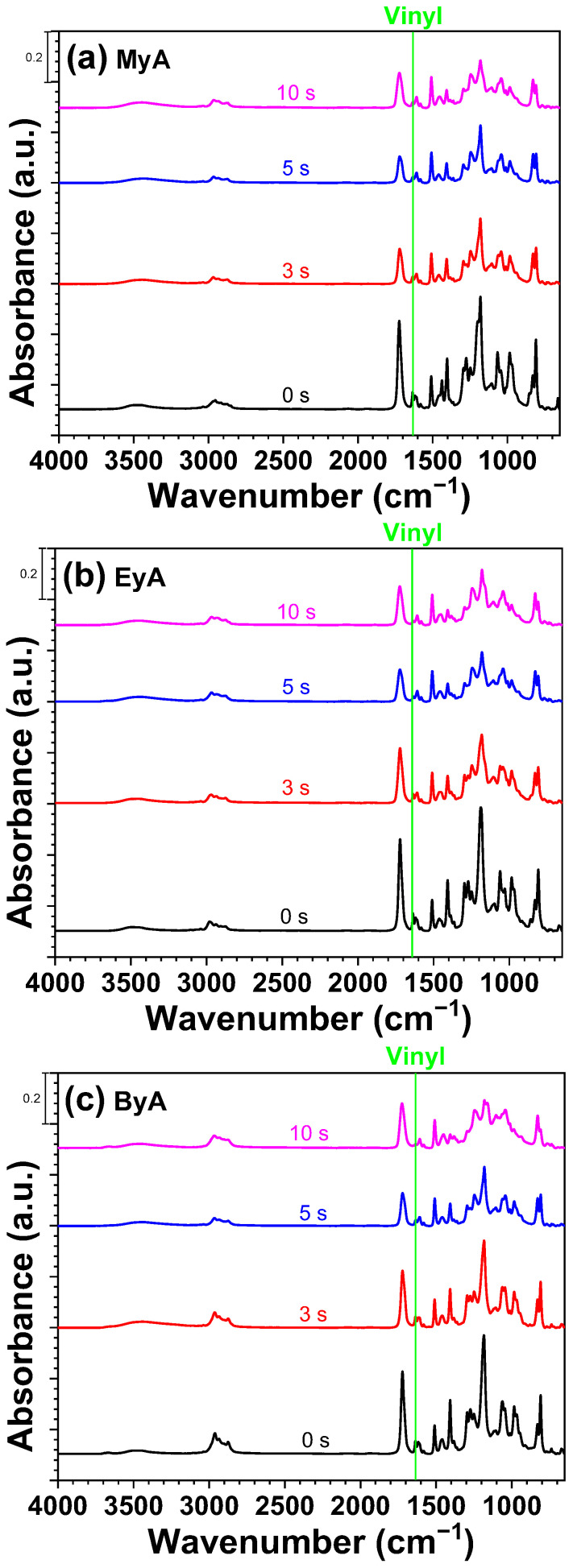
FTIR spectra of UV-curable resins with photoinitiator (HMPP) as a function of UV curing time (0, 3, 5, and 10 s).

**Figure 8:**
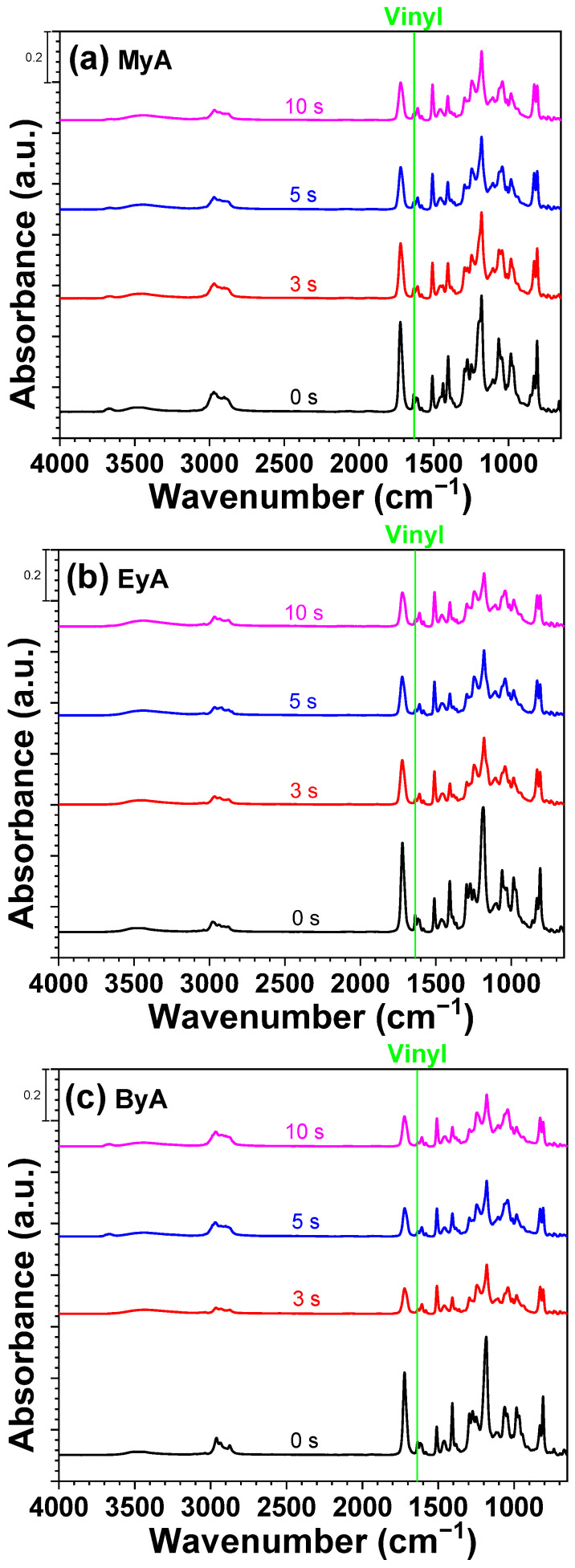
FTIR spectra of UV-curable resins with photoinitiator (TPO) as a function of UV curing time (0, 3, 5, and 10 s).

**Figure 9:**
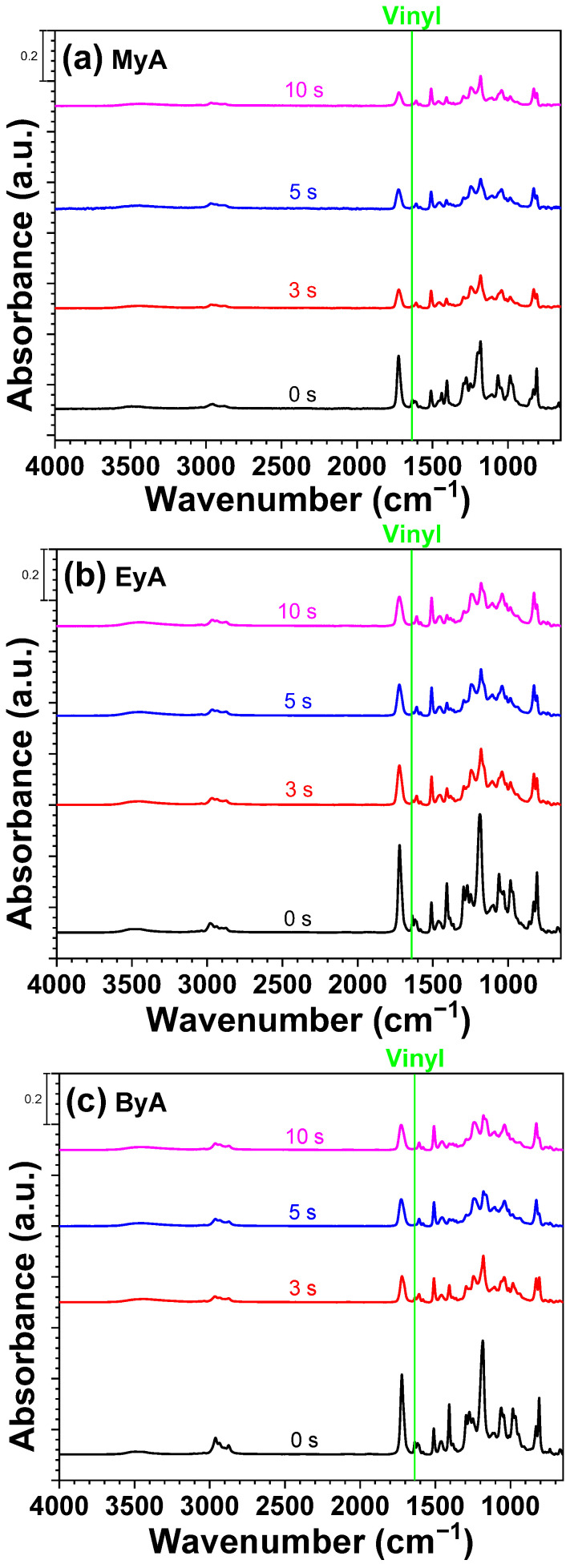
FTIR spectra of UV-curable resins with photoinitiator (MIX) as a function of UV curing time (0, 3, 5, and 10 s).

FT-IR spectroscopy was employed to monitor the photocuring process and to assess whether complete curing was achieved, as illustrated in Figure 7, Figure 8 and Figure 9. The photocuring reaction proceeds through a free-radical mechanism, in which vinyl double bonds (C=C) in the epoxy acrylate and curing agents form cross-linked networks [38]. During this process, not all vinyl groups may react, and the remaining unreacted groups are referred to as residual double bonds.

The FT-IR analysis specifically focused on the absorption band in the range of 1680–1620 cm^−1^, corresponding to the stretching vibrations of vinyl groups. These peaks diminished as the curing reaction progressed, indicating the consumption of reactive double bonds. After 5 s of UV exposure, the peak intensity decreased by approximately 50%, suggesting that a substantial portion of the polymerization occurred within the initial stages of irradiation. No significant further decrease was observed at 10 s, implying that the reaction either reached completion or that radical propagation slowed due to reduced radical and molecular mobilities (physical vitrification) or monomer depletion especially near the surface. This behavior is consistent with typical radical curing systems, where the highest rate of reaction occurs in the early stages before the system vitrifies or becomes diffusion-limited.

**Figure 10:**
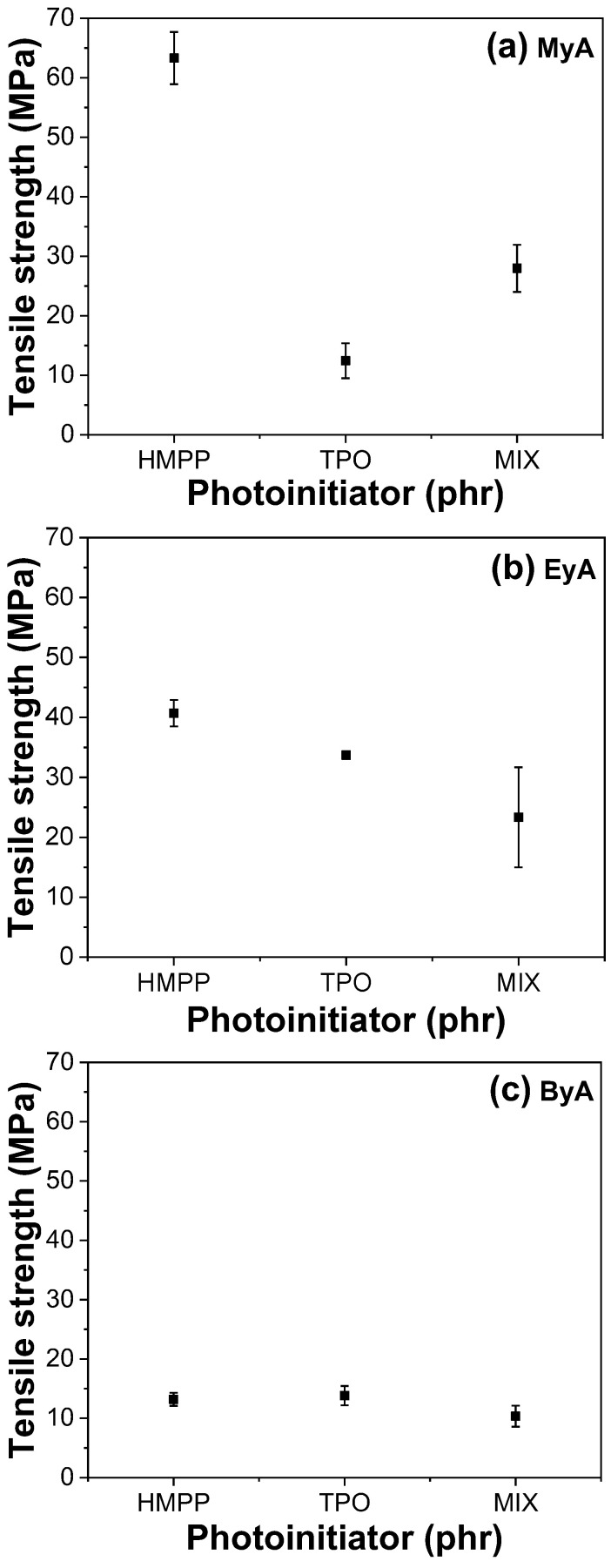
Tensile strengths of UV-cured samples (10 s) with different curing agents and photoinitiators.

**Figure 11:**
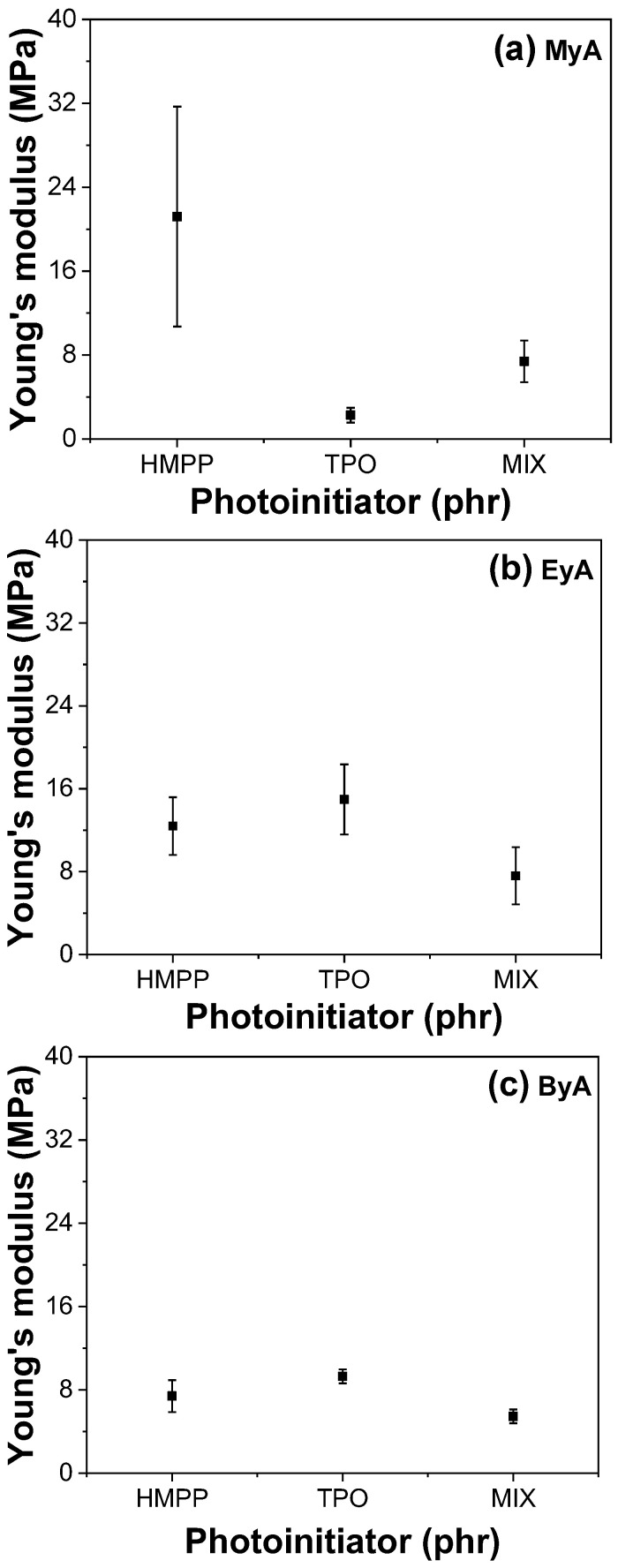
Young’s modulus of UV-cured samples (10 s) with different curing agents and photoinitiators.

**Figure 12:**
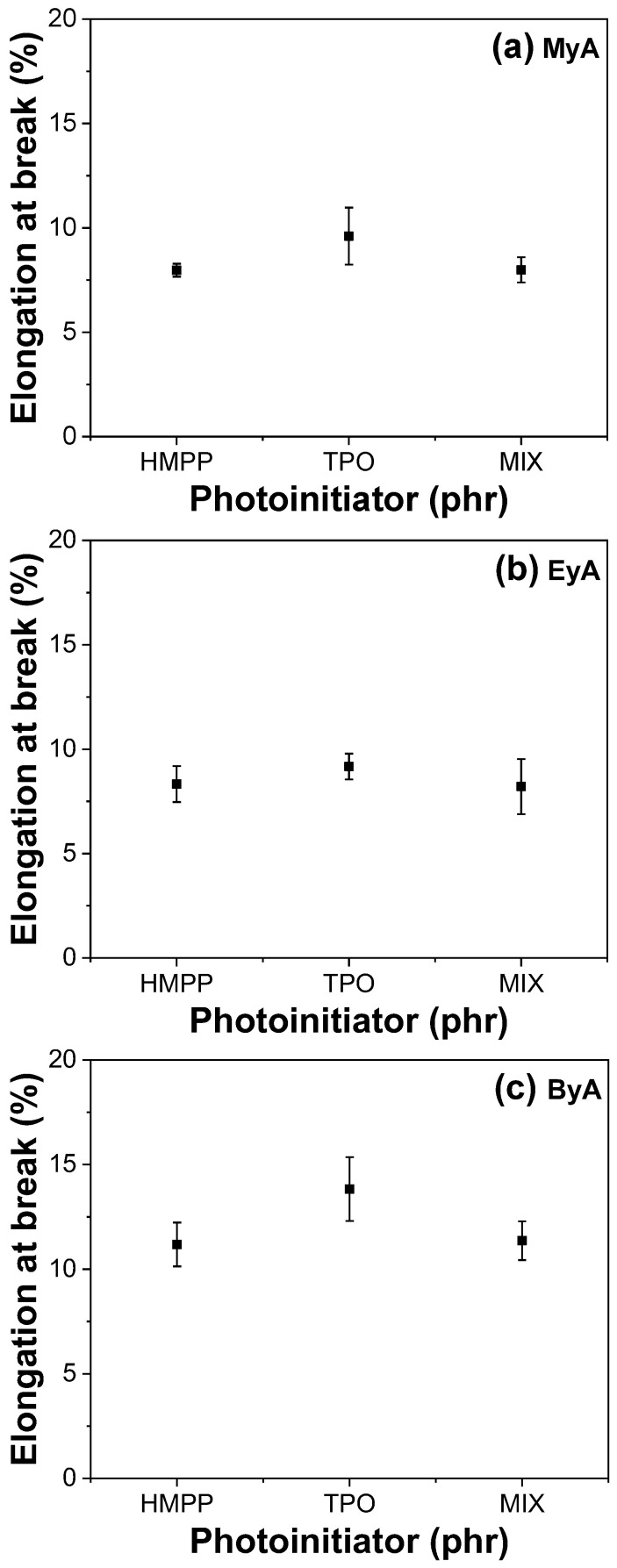
Elongation at break of UV-cured samples (10 s) with different curing agents and photoinitiators.

**Figure 13:**
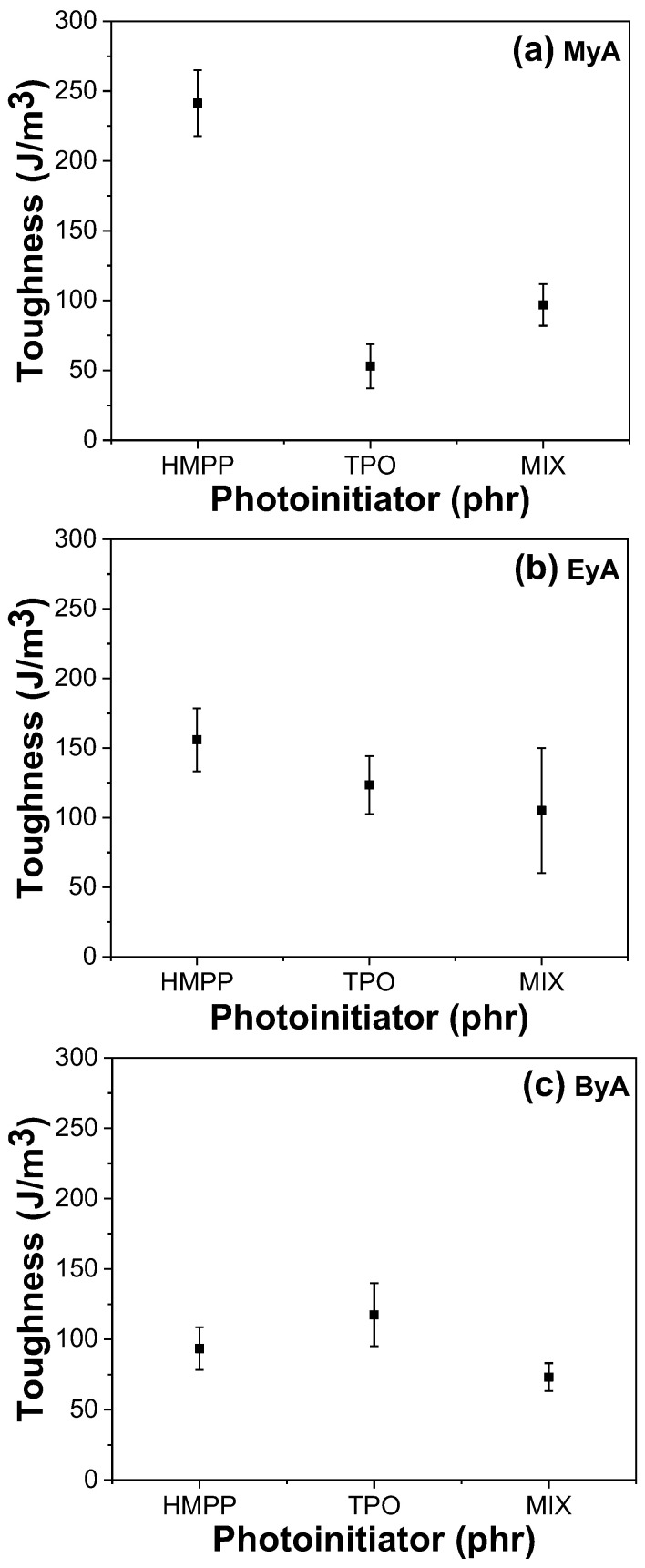
Toughness of UV-cured samples (10 s) with different curing agents and photoinitiators.

The reduction in vinyl group intensity correlates with the curing time and is reflected in Figure 10, Figure 11, Figure 12 and Figure 13. In addition, visual inspection of the samples revealed incomplete curing in the formulation containing ByA as the curing agent, as shown in Figure 5c. This aligns with the FT-IR data presented in Figure 5a,b, where the rate of vinyl group consumption in the ByA-based system was notably slower than in systems using MyA or EyA. The long, flexible alkyl chain of ByA likely hinders efficient network formation by reducing monomer reactivity and increasing chain mobility, leading to lower crosslink density and delayed vinyl group conversion. These findings highlight the direct relationship between chemical structure, reactivity, and curing efficiency in UV-initiated systems.

### 3.4. Mechanical Properties of Cured UV Sample

Photocuring significantly enhances the mechanical properties of polymeric materials by forming a 3D crosslinked network that provides high tensile strength, increased hardness, chemical resistance, thermal stability, and rapid curing. However, achieving optimal performance depends heavily on parameters such as the type of curing agent and photoinitiator, as well as curing time. Figure 10, Figure 11, Figure 12 and Figure 13 present the mechanical performance of various UV-cured formulations. Among the tested formulations, samples using ByA as the curing agent exhibited the lowest mechanical performance. This result is consistent with the visual observations in Figure 5c, where incomplete surface curing was evident. The long hydrocarbon side chain of ByA increases chain flexibility and free volume, which reduces the degree of crosslinking and thus lowers tensile strength and Young’s modulus. While flexibility can sometimes improve elongation at break, the ByA-based samples also showed limited elongation and low toughness, defined as the total energy absorbed before fracture, indicating a soft yet mechanically weak network. In addition, the influence of photoinitiator type was minimal in ByA-based systems, likely due to the dominant effect of the curing agent’s structure. This trend can also be confirmed in the thermal (DSC) and thermal–mechanical (DMA will be shown) analysis, where both results showed lower T_g_ values, further indicating low crosslink density.

In contrast, the samples cured with MyA showed strong dependence on photoinitiator type. HMPP led to the highest tensile strength and modulus, attributed to its high radical generation efficiency and low activation energy, which allowed rapid and uniform polymerization with MyA. This resulted in a densely crosslinked network. The MIX photoinitiator produced slightly lower mechanical properties, possibly owing to kinetic mismatch or uneven radical formation during curing. Notably, the MyA–TPO combination resulted in the lowest mechanical performance in this series. Although TPO is a highly efficient photoinitiator in many systems, its slower or spatially limited radical production may have caused insufficient or delayed crosslinking with MyA. This is supported by the low T_g_ values observed in DMA results, indicating incomplete network formation. Similarly, when EyA was used as the curing agent, the lowest mechanical properties were observed in samples cured with the MIX photoinitiator. The likely cause is inconsistent radical generation from the combination of HMPP and TPO, which may have led to partial curing and lower network density. This interpretation is further supported by thermal data, as the T_g_ value for the MIX–EyA system in the DMA results was also the lowest among the EyA samples.

Overall, HMPP was the most effective photoinitiator for producing robust and mechanically strong networks across different curing agents. In contrast, TPO and the MIX system were less effective, especially in formulations where rapid and complete curing was critical. These findings highlight the importance of matching photoinitiator reactivity with the curing agent structure to optimize both curing efficiency and mechanical performance [39].

DMA is a powerful tool for evaluating the mechanical behavior of UV-cured and UV-curable materials as a function of temperature, particularly for determining the T_g_. Various methods are available for determination of T_g_ using DMA, including the peak of the loss modulus (E″), the peak of tan δ, and the onset or drop in storage modulus (E′). In this study, T_g_ was identified from the temperature at which the loss modulus reached its maximum, as the tan δ curves displayed multiple peaks, suggesting complex relaxation behavior possibly due to heterogeneous crosslink density, phase separation from incompatible components, such as TPO + ByA, side reactions, vitrification trapping unreacted species, or partial curing.

**Figure 14:**
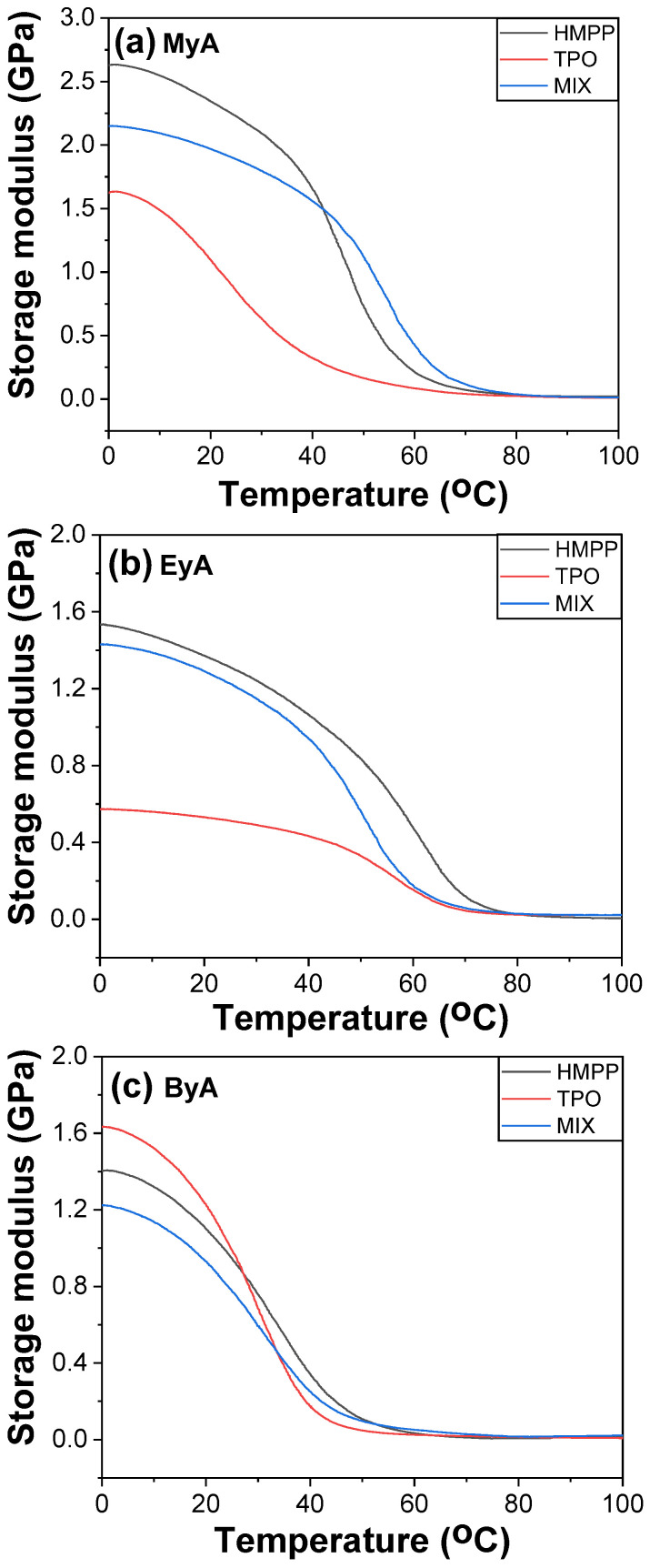
Storage modulus of UV-cured samples (10 s) with different curing agents and photoinitiators.

**Figure 15:**
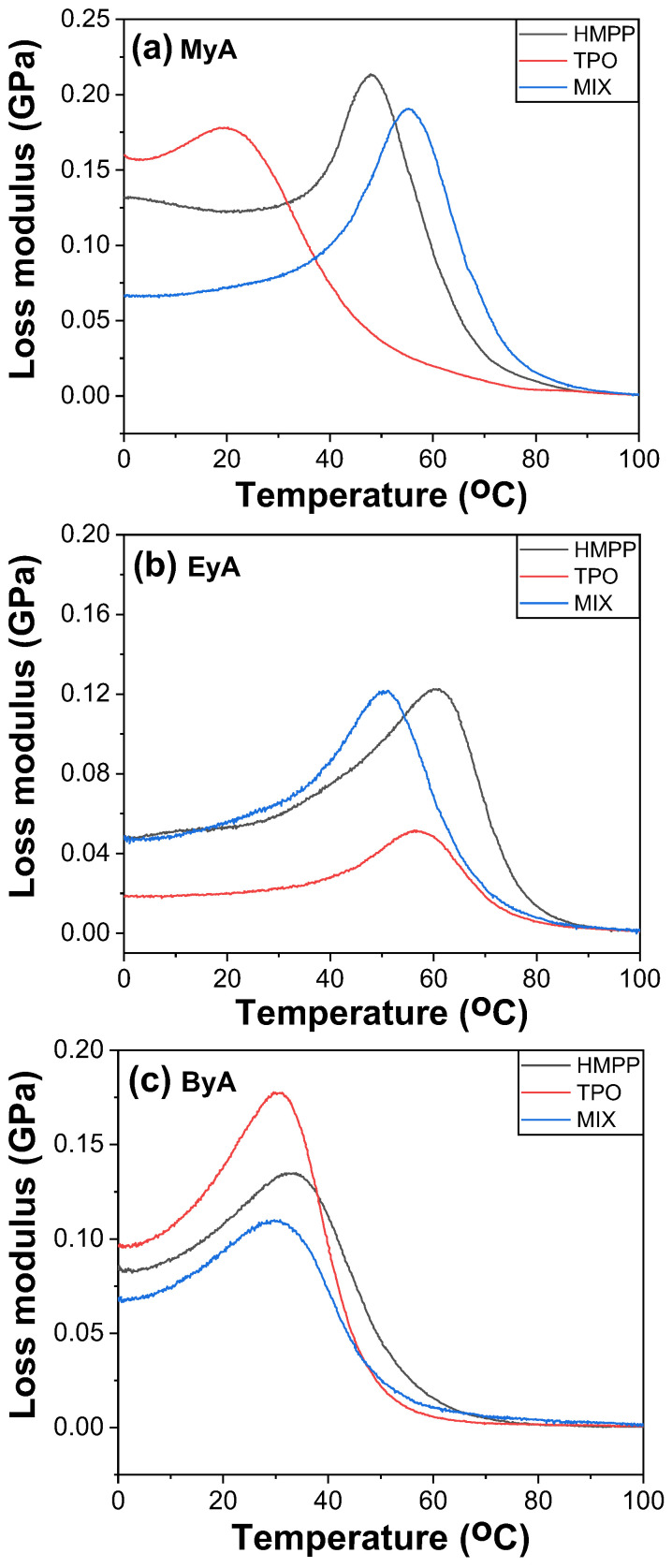
Loss modulus of UV-cured samples (10 s) with different curing agents and photoinitiators.

**Figure 16:**
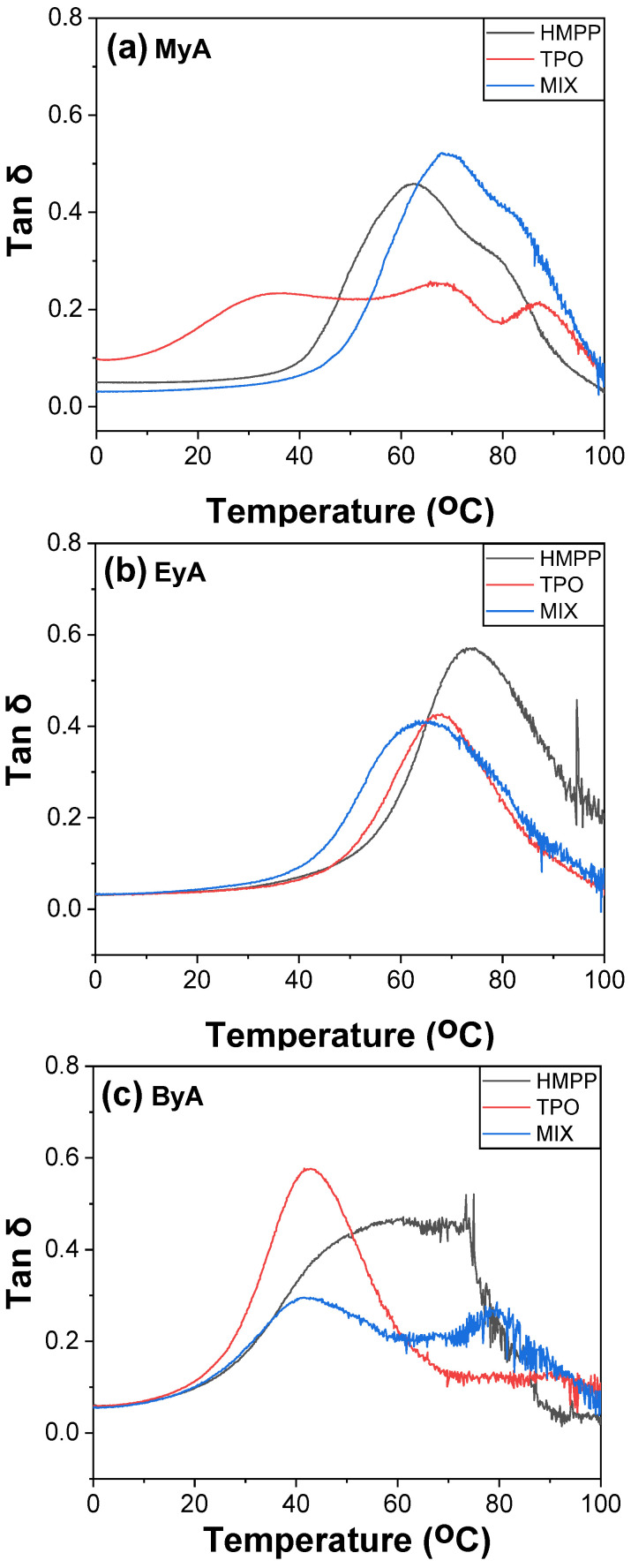
Tan δ of UV-cured samples (10 s) with different curing agents and photoinitiators.

Figure 14, Figure 15 and Figure 16 display the storage modulus, loss modulus, and tan δ of the UV-cured samples. The measured T_g_ values were generally consistent with those obtained from mDSC, demonstrating reliable cross-validation between thermal and mechanical analyses. However, samples cured with TPO showed a noticeable deviation, with lower T_g_ values observed in DMA than in DSC. This is because DSC reflects the average thermal transition based on a thermogram, whereas DMA monitors the mechanical response. This difference implies that whereas TPO enables efficient crosslinking and elevates bulk thermal properties (as captured by DSC), it may introduce mechanical inhomogeneities, uncured domains, local defects, or localized brittleness that reduce the effective T_g_ under dynamic loading conditions. This supports the interpretation that high reactivity of TPO may lead to excessively fast or uneven curing, creating network imperfections that negatively impact the mechanical integrity of the polymer.

Furthermore, the storage modulus values obtained from DMA showed strong agreement with the Young’s modulus data from tensile testing (UTM), indicating consistent trends in stiffness across both mechanical testing methods. This correlation reinforces the reliability of the DMA results and supports the conclusion that photoinitiator selection, especially the use of TPO, plays a critical role not only in curing efficiency but also in the mechanical uniformity and final performance of the cured network.

In summary, the lower T_g_ values observed via DMA in TPO-cured samples provide strong evidence that overly rapid or uneven crosslinking can compromise mechanical performance, even when thermal analysis suggests full conversion. This highlights the importance of balancing curing speed with network uniformity when selecting photoinitiators for high-performance UV-curable systems.

## 4. Conclusions

This study demonstrated the critical role of photoinitiators in determining the thermal, mechanical, and morphological properties of UV-curable epoxy acrylate systems. Among the tested photoinitiators, HMPP exhibited superior performance by generating strong cross-linked networks, resulting in high tensile strength, stiffness, and improved fracture morphology. HMPP-cured samples (MyA-HMPP) showed a T_g_ of 67.0 °C and tensile strength of 63.3 MPa at a UV curing time of 10 s. TPO demonstrated excellent thermal properties but was less effective in achieving optimal mechanical performance. TPO-cured samples (MyA-TPO) showed a T_g_ of 75.6 °C and tensile strength of 12.5 MPa at a UV curing time of 10 s. The MIX photoinitiator exhibited intermediate characteristics, with slower or incomplete curing observed in some cases. Thermal and FT-IR analyses confirmed that curing time significantly impacted the T_g_ and residual double bond conversion, indicating the necessity of optimizing curing parameters for different formulations. SEM imaging further validated the influence of photoinitiators on fracture characteristics and mechanical integrity. These findings reveal the importance of photoinitiator selection and system design for high-performance UV-curable materials, particularly for applications in advanced packaging and electronics. Future work could explore the integration of additional additives and varied curing light sources to enhance long-term stability and application-specific performance.

## Figures and Tables

**Table 1:** Additive content of UV curing paste.

Name	EA	Curing Agent (1:1 Equivalent Ratio)	Photoinitiator (HMPP/TPO/MIX)
ByA	5 g	4.05 g	0.09 g
MyA		2.72 g	0.08 g
EyA		3.17 g	0.08 g

## Data Availability

The data presented in this study are available on request. Further inquiries can be directed to the corresponding author.

## Supplementary Materials

The following supporting information can be downloaded at: https://www.mdpi.com/article/10.3390/polym17091252/s1, Figure S1: SEM images of fracture surfaces of UV-cured samples with various curing agents and photoinitiators.
