# Supplementary material for: Interactions and Curing Dynamics Between UV-Triggered Epoxy Acrylate Binder, Curing Agents and Photoinitiators
Source: Polymers (Basel). 2025 May 4;17(9):1252. doi: 10.3390/polym17091252 (PMC12073225; doi:10.3390/polym17091252)
Supplement: Supplementary file 1 [file polymers-17-01252-s001.zip › polymers-3583710-supplementary.pdf]

Interactions and Curing Dynamics between UV-triggered Epoxy Acrylate Binder, Curing Agents and Photoinitiators

*Ji-min Choi [1], Sang Jang[1], and Keon-Soo Jang**

Department of Polymer Engineering, School of Chemical and Materials Engineering,

The University of Suwon, Hwaseong, Gyeonggi-do,18323, Republic of Korea

[1]: These authors (J. Choi and S. Jang) contributed equally: Co-1st authors

CORRESPONDING AUTHOR FOOTNOTE *To whom correspondence should be addressed. K.-S. Jang: ksjang@suwon.ac.kr

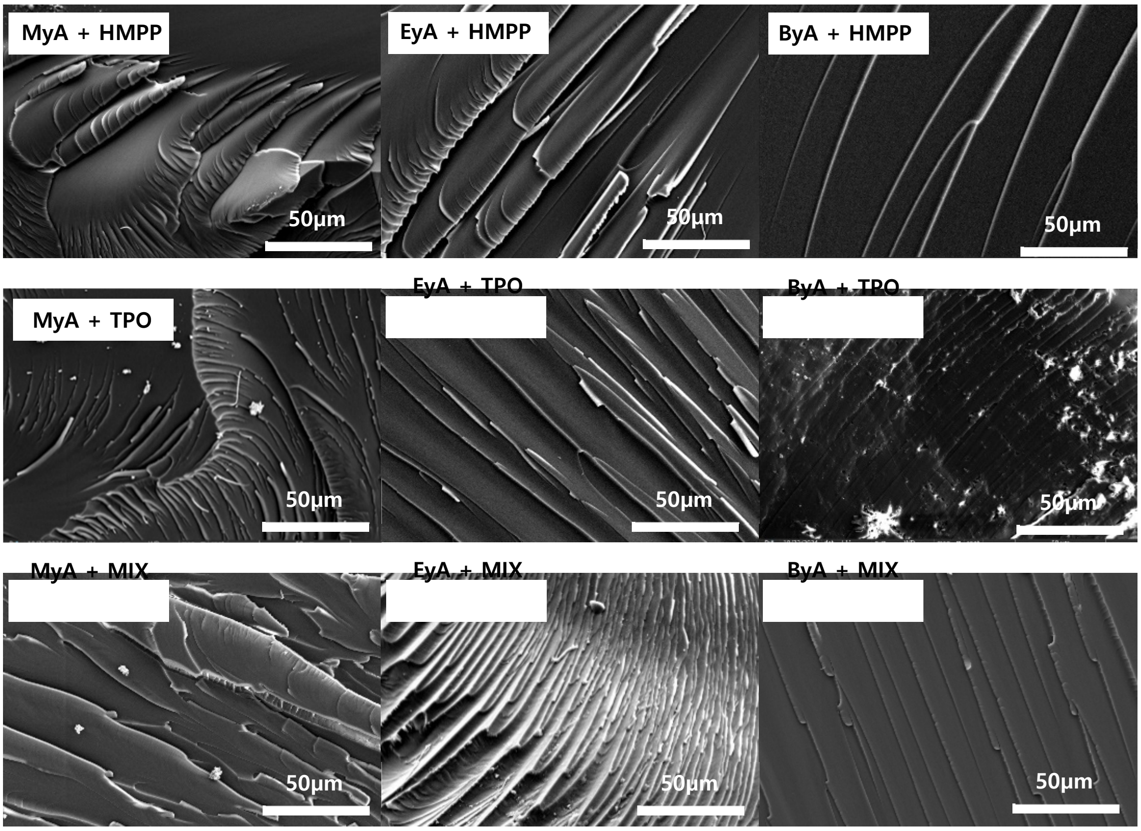

Figure S1. SEM images of fracture surfaces of UV-cured samples with various curing agents and photoinitiators.
